# Performance of Deep Learning-Based Algorithm for Detection of Pediatric Intussusception on Abdominal Ultrasound Images

**DOI:** 10.1155/2022/9285238

**Published:** 2022-08-12

**Authors:** Zheming Li, Chunze Song, Jian Huang, Jing Li, Shoujiang Huang, Baoxin Qian, Xing Chen, Shasha Hu, Ting Shu, Gang Yu

**Affiliations:** ^1^Department of Data and Information, The Children's Hospital Zhejiang University School of Medicine, Hangzhou 310052, China; ^2^Sino-Finland Joint AI Laboratory for Child Health of Zhejiang Province, China; ^3^National Clinical Research Center for Child Health, Hangzhou, China; ^4^Polytechnic Institute, Zhejiang University, 866 Yuhangtang Rd, Hangzhou 310058, China; ^5^Department of Ultrasound, The Children's Hospital Zhejiang University School of Medicine, Hangzhou 310052, China; ^6^Department of Day Surgery, The Children's Hospital Zhejiang University School of Medicine, Hangzhou 310052, China; ^7^Huiying Medical Technology (Beijing), Beijing 100192, China; ^8^Hangzhou Normal University, 310052 Hangzhou, China; ^9^The Children's Hospital Zhejiang University School of Medicine, Hangzhou 310052, China; ^10^National Institute of Hospital Administration, NHC, Beijing 100044, China

## Abstract

**Background and Aims:**

Diagnosing pediatric intussusception from ultrasound images can be a difficult task in many primary care hospitals that lack experienced radiologists. To address this challenge, this study developed an artificial intelligence- (AI-) based system for automatic detection of “concentric circles” signs on ultrasound images, thereby improving the efficiency and accuracy of pediatric intussusception diagnosis.

**Methods:**

A total of 440 cases (373 pediatric intussusception and 67 normal cases) were retrospectively collected from Children's Hospital affiliated to Zhejiang University School of Medicine from January 2020 to December 2020. An improved Faster RCNN deep learning framework was used to detect “concentric circle” signs. Finally, independent validation set was used to evaluate the performance of the developed AI tool.

**Results:**

The data of pediatric intussusception were divided into a training set and validation set according to the ratio of 8 : 2, with training set (298 pediatric intussusception) and validation set (75 pediatric intussusception and 67 normal cases). In the “concentric circle” detection model, the detection rate, recall, specificity, and *F*1 score assessed by the validation set were 92.8%, 95.0%, 92.2%, and 86.4%, respectively. Pediatric intussusception was classified by “concentric circle” signs, and the accuracy, recall, specificity, and *F*1 score were 93.0%, 92.0%, 94.1%, and 93.2% on the validation set, respectively.

**Conclusion:**

The model established in this paper can realize the automatic detection of “concentric circle” signs in the ultrasound images of abdominal intussusception in children; the AI tool can improve the diagnosis speed of pediatric intussusception. It is necessary to further develop an artificial intelligence system for real-time detection of “concentric circles” in ultrasound images for the judgment of children with intussusception.

## 1. Introduction

Intussusception is a kind of pediatric surgical acute abdomen, which is relatively common in clinic and mainly affects children under 2 years old [1–3]. Among the high number of pediatric emergency abdominal patients, the average annual incidence of intussusception in children in China is 418.1/100,000 [4, 5], which has exceeded the global average of 74/100,000 [6]. Intussusception mainly refers to the phenomenon of interlocking two intestinal tubes, early and timely diagnosis and active and correct treatment can prevent intestinal necrosis and relieve the pain of children [7, 8]. Ultrasound (US) as a noninvasive and painless examination method is easy to be accepted by children and their families [9, 10]. The ultrasound images of typical children intussusception can be summarized as two signs [11]: one is the “concentric circle” sign on the cross section, and the other is the “sleeve sign” sign on the vertical section. Doctors mostly judge whether patients have intussusception problem by identifying the “concentric circle” sign [12]. However, the increasing ultrasound image data also brings burden to doctors' diagnosis. In recent years, with the depth of the study in the field of deep learning applications, deep learning technology is often used in the field of fast and intelligent image processing, such as image classification and detection. Deep learning simulates human vision mechanism, and has the advantages of fast detection speed and low cost, especially in the field of medical imaging has made breakthrough progress. Many studies have shown that AI achieved surprising results. Qian et al. [13] developed an explainable deep-learning system based on multimodal breast-ultrasound images and predicted BI-RADS scores for breast cancer as accurately as experienced radiologists. Sui et al. [14] developed a deep-learning AI model (ThyNet) to differentiate between malignant tumours and benign thyroid nodules and aimed to investigate how ThyNet could help radiologists improve diagnostic performance and avoid unnecessary fine needle aspiration. Tiyarattanachai et al. [15] developed a deep learning network for the detection and diagnosis of focal liver lesions from ultrasound images, AI model detected and diagnosed common focal liver lesions. For diagnosis of hepatocellular carcinomas, the AI model yielded sensitivity, specificity, and negative predictive value of 73.6%, 97.8%, and 96.5% on the internal validation set. Although artificial intelligence is widely used in the detection and classification of lesions in breast, thyroid, liver and other ultrasound images, the application of artificial intelligence in pediatrics is still in its infancy.

Region Convolutional Neural Network features (RCNN) with convolutional neural network features were proposed for target detection by Girshick et al. [16] in 2014. Since then, target detection has started to evolve at an unprecedented rate. Although RCNN has made great progress, it requires a large amount of redundant feature computation, resulting in extremely slow detection. In 2015, Girshick proposed the Fast RCNN detector [17], which is a further improvement to RCNN. Fast RCNN allows us to train both the detector and the bounding box regressor in the same network configuration. The detection speed is more than 200 times faster than that of RCNN. Although Fast-RCNN successfully computes the feature mapping only once for the whole image, its detection speed is still limited by region proposal network. In 2015, Ren et al. proposed the Faster RCNN detector [18], which is the first end-to-end and the first near real-time deep learning detector. Since then Faster RCNNs have been widely used for detection tasks.

Inspired by the classical detection network Faster RCNN, this paper developed a model more suitable for detecting “concentric circles” in ultrasound images. To the best of our knowledge, this is the first attempt that makes use of deep learning to diagnose intussusception in children, it makes up for the blank of using deep learning for diagnosing pediatric intussusception based on ultrasound images.

## 2. Material and Method

The retrospective study was approved by institutional ethics committee of Children's Hospital Affiliated to Zhejiang University School, and a waiver for informed consent was provided.

### 2.1. Patient Selection

#### 2.1.1. Selection Criteria

1. Patients diagnosed with intussusception and normal cases
2. The abdominal ultrasound image data were complete
3. Ultrasound images are clear

#### 2.1.2. Exclusion Criteria

1. Previous abdominal surgery
2. There are motion artifacts and foreign bodies in the ultrasound image

This study retrospectively collected the images and clinical data of pediatric intussusception in admitted to Children's Hospital Affiliated to Zhejiang University School of Medicine from January 2020 to December 2020. A total of 440 children were included in this study, with 372 pediatric intussusception and 67 normal children.

### 2.2. US Image Acquisition

With ultrasound examination instruments and equipment examination methods: Philip IE33 and iuEilite color ultrasound instrument used linear array probe frequency *L* 5~12 and convex array probe frequency 1~5 MHz, the child was in supine position, with a linear array probe combined with a convex array probe for detailed scan of the entire abdominal bowel. After the lesions were found, multisection scanning, such as longitudinal and transverse resection, was performed to observe the lesions in real time with the change of body position of the children. After the lesions were clearly displayed, the location of the mass was recorded, the diameter of the “concentric circle “ sign and the length of the “sleeve sign” were measured, and the mesenteric lymph nodes in the lesions were observed.

**Figure 1:**
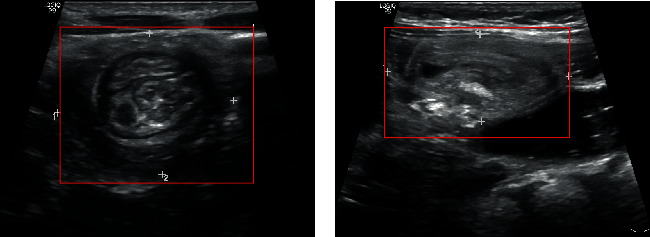
Signs of “concentric circles” at different angles. A child diagnosed with intussusception (female, 2 years old) presents with abdominal pain, vomiting, hematochezia, and abdominal mass. The “concentric circle” area is marked with a white cross: (a) easy to observe and (b) hard to observe.

The echogram of pediatric intussusception showed “concentric circle” signs. Signs of “concentric circles” at different angles are shown in Figure 1. For naked eye observation, some “concentric circle” signs are obvious and easy to observe, such as Figure 1(a). However, the image comparison between the target area and background area is too low and the edge is blurred, and the “concentric circle” signs are irregular, such as Figure 1(b); these characteristics are also difficult to be recognized by human experts, so the detection of “concentric circle” signs is a challenging task.

### 2.3. Data Processing

All data were static images taken by the subjects during ultrasound, and the images were stored in DICOM format. The dataset consists of images from different scales, with an average image size of 1341 × 864 pixels. Because of the subjectivity of probe strafing, some of these images contain “concentric circle” signs, while others do not. In the process of image preprocessing, all the subject identification information and peripheral regions in the ultrasound images are cropped to ensure that the cropped image only contains the fan-shaped ultrasound region.

### 2.4. AI Model Development

**Figure 2:**
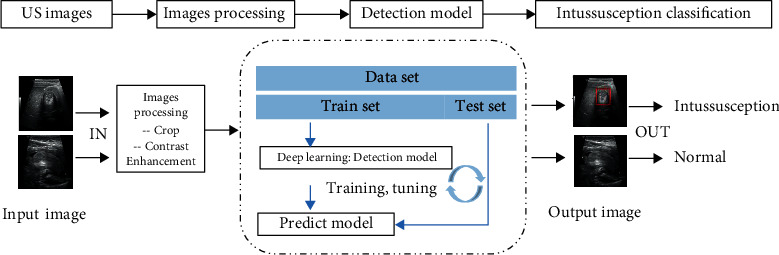
Overall flow chart.

This study is divided into two parts: the first part includes image preprocessing and detection of “concentric circle” signs using an improved Faster RCNN network; the second part is based on the detection of “concentric circle” signs to complete the classification of pediatric intussusception and normal cases. The overall flow chart of this study is shown in Figure 2. The original DICOM images were first cropped and contrast enhanced; then, the images after processing were fed into the detection model for training, and based on the model's prediction of the “concentric circle” signs, the final category and its probabilities were predicted to give a higher category probability and a prediction score for patients with intussusception.

### 2.5. Design of the Detection Model

**Figure 3:**
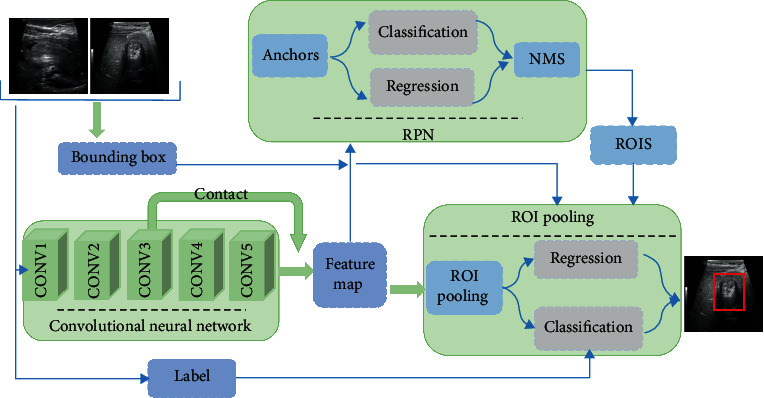
Network structure. The main structure of the network consists of three stages: backbone for feature extraction network, region proposal network (RPN), and region of interest (ROI) pooling. Input the image into the VGG16 to get the feature map, use RPN to generate the anchors, after Nonmaximum Suppression (NMS) to obtain ROIs, project the ROIs onto the feature map to get the feature matrix, scale each feature matrix to 7 × 7 through the ROI pooling, and then flatten the feature map to get the prediction result through a series of fully connected layers.

Inspired by the classical detection network Faster RCNN, this paper developed a model more suitable for detecting “concentric circle” signs in ultrasound images. In order to distinguish standard “concentric circles” and nonstandard “concentric circles” better, this paper adds a jump connection to the convolutional neural network. By combining the shallow and deep features of the convolutional neural network with the jumping connection layer, the detector can detect the regions with insignificant background differences and no obvious “concentric circles” signs. Based on the Faster RCNN network model, this paper uses layer connection to connect the features collected at conv3 and conv5 layers of VGG16 [19] (network structure is shown in Figure 3), so as to fuse shallow information and deep features to better mine semantic features in images. We changed the last fully connected layer to predict two categories: the “concentric circle” area and the background. In addition, we retrain the last fully connected layer and calculate the coordinates and confidence of the “concentric circle” region and background.

### 2.6. Experiments

**Figure 4:**
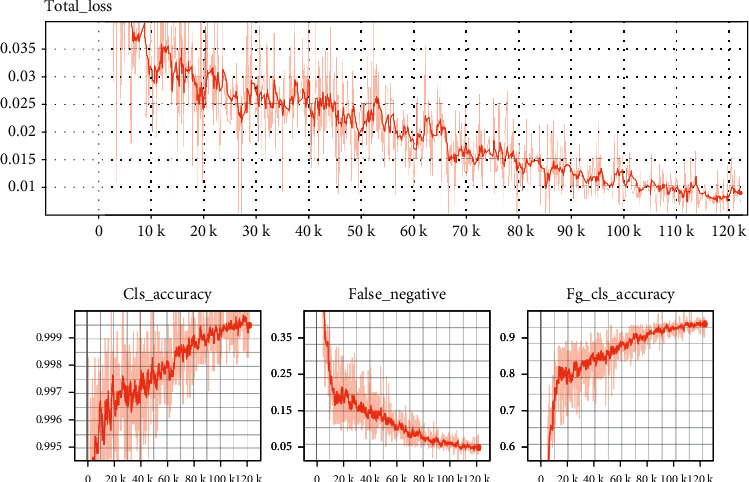
Training details. (a) Loss curves of training sets under different iterations and (b) variation curves of different evaluation index values of training sets under different epochs.

The model was trained by the method of supervision training. To obtain the corresponding image label, the position of the “concentric circle” signs in the ultrasound image was traced by an experienced sonographer and verified by another expert label to ensure accuracy. We used PyTorch framework to train the model for 120 iterations (120 epochs, each with 1000 iterations) on 2 GeForce RTX 1080 Ti GPUs. For model training, the Batch size was set to 64, and the initial learning rate was set to 0.01. Use warm restart learning rate to adjust learning rate [20]. Cosine function can be used to reduce learning rate. In the cosine function, with the increase in *x*, the cosine value decreases slowly at first, then accelerates, and slowly decreases again. This kind of decline mode can be combined with the learning rate to produce good results in a very effective way of calculation. The training time was 1 D 0 H 24 min. The loss curve of model training is shown in Figure 4(a). The loss curve shows that when the number of iterations is 120,000, the model is in the fitting state. On the other hand, the model is in an ideal training state when 120,000 iterations are reached. The model with 120,000 training times was selected as the final detector for testing.  Figure 4(b) shows that with the increase in epoch times, each evaluation index tends to be stable.

### 2.7. Statistical Analysis

SPSS 22.0 was used to identify differences in clinical features between patients with intussusception and the normal case group. Continuous variables were expressed as mean ± standard deviation, and the two-sided Student *t*-test was used to compare whether there were significant differences between groups. Discrete variables were expressed as counts (percentages), and Pearson's chi-square test was used. If *P* < 0.05, the variables were considered to be significantly different among different groups.

## 3. Results

### 3.1. Clinical Characteristics

**Figure 5:**
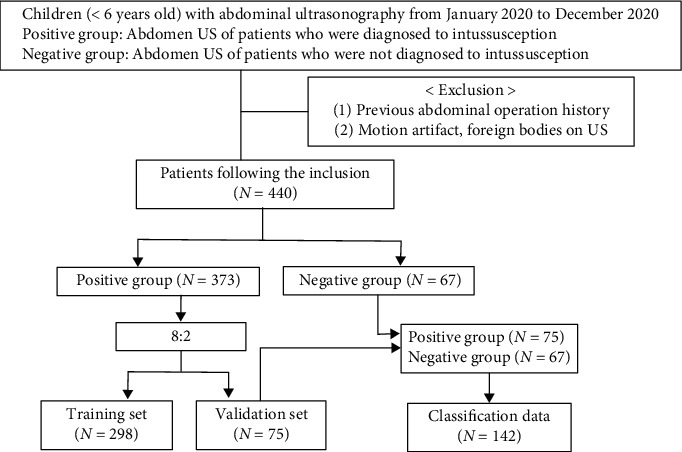
Flow chart of data collection.

According to the criteria, 440 patients (306 males and 134 females, mean age 33.9 ± 29.1 months) were enrolled. The flow chart for data collection is shown in Figure 5.

A total of 440 patients were included in this study, including 373 patients with intussusception (male : female = 266 : 107, mean age: 28.6 ± 21.5 months) and 67 patients in the normal group (male : female = 40 : 27, mean age: 39.1 ± 36.6 months).  Table 1 shows the demographics of the two groups. Univariate analysis of age and sex by independent sample *t*-test and Pearson's chi-square test showed that the patients with intussusception were younger than normal in demographic characteristics (*P* = 0.001). The majority of intussusception patients were boys (*P* < 0.001), and the results were statistically significant.

### 3.2. Data Split

A total of 440 cases were included in this study, with 373 pediatric intussusception patients and 67 children. There were 5 to 10 ultrasound images in each case, no ultrasound images with “concentric circle” signs in the normal group, and 1 to 3 ultrasound images with “concentric circle” signs in each intussusception patient, and a total of 715 ultrasound images with “concentric circle” signs were labeled. The data set was divided by 8 : 2, with 80% of the data as a training set (2325 images of 298 pediatric intussusception patients, including 575 positive samples); 20% of the data (586 images of 75 pediatric intussusception patients, including 140 positive samples) were used as a validation set to evaluate the performance of the concentric circle signature detection model. 75 pediatric intussusception patients and 67 normal cases were classified to evaluate the generalization ability of the model (for detection data, see Table 2, and for classification data, see Table 3).

### 3.3. Performance and Evaluation of the Detection Model

The detection rate of the “concentric circle” signs in each image was evaluated: if the prediction model generated a boundary box around the image and the box overlapped with the real location of the “concentric circle” signs, the “concentric circles” were judged to be correctly detected. In this study, different confidence levels were used to evaluate the detection effect. The detection effect of concentric circles varies with different confidence levels, as shown in Table 4. Accuracy (Acc) was calculated by dividing the true positive (TP) number of correctly detected concentric circle signs by the total number of concentric circle signs. When the model outputs a bounding box in an area that does not contain a “concentric circle” sign, the count is false positive (FP). Evaluation indicators are defined as follows:

(1) $$\begin{array}{c} \text{Acc}=\frac{\text{TP}+\text{TN}}{\text{TP}+\text{TN}+\text{FP}+\text{FN}},\\ \text{Spe}=\frac{\text{TN}}{\text{TN}+\text{FP}},\\ \text{Recall}=\frac{\text{TP}}{\text{TP}+\text{FN}},\\ \text{Precision}=\frac{\text{TP}}{\text{TP}+\text{FP}},\\ F1\text{ score}=2\times \frac{\text{precision}\times \text{recall}}{\text{precision}+\text{recall}}. \end{array}$$

**Figure 6:**
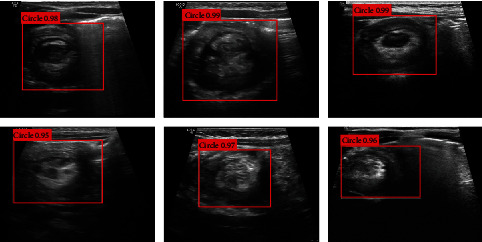
Ultrasound images of pediatric intussusception in the validation set.

Finally, 0.5 was used as the cut-off point of confidence threshold. In the “concentric circle” sign detection model, the detection rate, recall rate, specificity, and *F*1 score evaluated by the validation set were 92.8%, 95.0%, 92.2% and 86.4%, respectively. Ultrasound images of pediatric intussusception in the validation data are shown in Figure 6.

**Figure 7:**
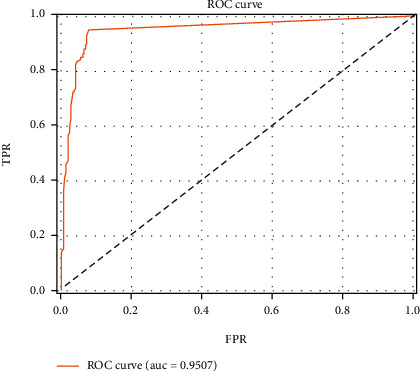
ROC curve of detection results on the validation set.

ROC curve of the final “concentric circles” sign detection model on the validation set is shown in Figure 7. The closer the ROC curve is to the upper left, the better the classifier performs. The Area under the Curve (AUC) of the “concentric circle” detection results on the validation set in this paper was 95.1%. After obtaining the ROC curve of the final “concentric circles” sign detection model, we determined an optimal threshold of 0.821 according to the maximized Youden index (sensitivity + specificity − 1) from the validation set.

### 3.4. Performance and Evaluation of Classification

**Figure 8:**
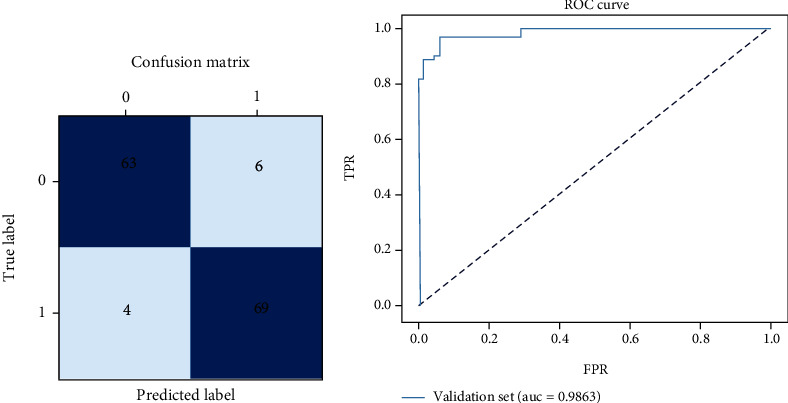
Classification results on the validation set. (a) Confusion matrix and (b) ROC curve.

Based on the results of the concentric circle detection, the algorithm used input images one by one to predict whether there was a dichotomy between normal cases and pediatric intussusception. The model gives the predicted pediatric intussusception score by multiplying the probability of an object's existence in ROI by the probability of category, and the class with higher probability is predicted as the final class and its probability. The validation set consisted of 75 pediatric intussusception and 67 normal cases. The confusion matrices on the validation set is presented in Figure 8(a). Where the number of true positive (TP), true negative (TN), false positive (FP), and false negative (FN) diagnoses were 69, 63, 6, and 4, respectively.  Table 5 shows the comparison of classification performance on the validation set using different evaluation indexes. The AUC, accuracy, recall, specificity, and *F*1 score were 98.6%, 93.0%, 92.0%, 94.1%, and 93.2%, respectively, on the validation set. ROC curve of classification results is shown in Figure 8(b). The AUC of pediatric intussusception diagnosis on the validation set was 98.6%. The results showed that the dichotomy of normal cases and pediatric intussusception had good classification performance according to the detection results of “concentric circle” signs.

## 4. Discussion

In this paper, an improved Faster RCNN was applied in the detection of “concentric circle” signs in abdominal ultrasound for the diagnosis of pediatric intussusception. The visualization results show that the model effectively learns the signs of concentric circles. The detection rate, recall, specificity, and *F*1 score of the algorithm evaluated in the validation set were 92.8%, 95.0%, 92.2%, and 86.4%, respectively. In our study, the algorithm used input images one by one to predict whether “concentric circle” signs exist to classify patients as normal cases and pediatric intussusception. The model through an object exists in the ROI probability and categories probability multiplication to give prediction score for pediatric intussusception. The class with the highest probability is predicted to be the final class and its probability. The accuracy, recall, specificity, and *F*1 score of the concentric circle signs in the diagnosis of pediatric intussusception were 93.0%, 92.0%, 94.1%, and 93.2% in the internal test set, respectively.

There are some differences between our study and previous studies. Kim et al. [21] developed and tested the performance of a deep learning-based algorithm to detect ileocolic intussusception using abdominal radiographs of young children, and a YOLOv3-based algorithm was developed to recognize the rectangular area of the right abdomen and to diagnose intussusception. The sensitivity of the algorithm was higher compared with that of the radiologists (0.76 vs. 0.46, *P* = 0.013). Compared with Kim et al.'s work, we used abdominal ultrasound images of children and the algorithm of Faster RCNN based on the detection of concentric circle signs to diagnose intussusception in children; the sensitivity of the algorithm is higher than that of Kim et al..

Our study also had certain limitations: (1) in this study of deep learning for the diagnosis of intussusception in children on abdominal ultrasound images, the amount of data used by our algorithm is limited. Future work will focus on improving the result of deep learning for the detection of intussusception by adding more training data. (2) The data for this paper came from only one hospital. In the future, data from multiple medical institutions can be considered for external validation to verify the generalization performance of the model. (3) The interpretability of the deep learning model has always been criticized. In the follow-up work, some interpretable features will be added to improve the interpretability of the model.

## 5. Conclusion

In summary, this paper has developed a deep learning framework for the detection of “concentric circle” signs on ultrasound images. The model has good accuracy and reliability for “concentric circle” detection; in addition, it has a high accuracy in classifying pediatric intussusception based on the results of the detection, and a chart review was conducted to confirm that the imaging correctly identified the diagnosis.

## Figures and Tables

**Table 1:** Patient baseline data.

	Intussusception	Normal	*P* value
All (*N* = 440)			
Patient, *n*	373	67	
Age (months), mean [SD]	28.6 [21.5]	39.1 [36.6]	0.001
Sex, male, *n* (%)	266 (71.3)	40 (59.7)	<0.001

**Table 2:** Division of detection data.

Detection data		Total case	Total images	Positive images	Negative images
Train set	Intussusception	298	2325	575	1750
Validation set	Intussusception	75	586	140	446
Total number		373	2911	715	2196

**Table 3:** Division of classification data.

	Intussusception	Normal	Total case
Classification data	75	67	142

**Table 4:** Comparison of evaluation index results under different confidence thresholds.

Threshold	TP	TN	FP	FN	Acc	Spe	Recall	Precision
0.0	133	411	35	7	92.8%	92.2%	95.0%	79.2%
0.05	133	411	35	7	92.8%	92.2%	95.0%	79.2%
0.10	133	411	35	7	92.8%	92.2%	95.0%	79.2%
0.15	133	411	35	7	92.8%	92.2%	95.0%	79.2%
0.20	133	411	35	7	92.8%	92.2%	95.0%	79.2%
0.25	133	411	35	7	92.8%	92.2%	95.0%	79.2%
0.30	133	411	35	7	92.8%	92.2%	95.0%	79.2%
0.35	133	411	35	7	92.8%	92.2%	95.0%	79.2%
0.40	133	411	35	7	92.8%	92.2%	95.0%	79.2%
0.45	133	411	35	7	92.8%	92.2%	95.0%	79.2%
0.50	133	411	35	7	92.8%	92.2%	95.0%	79.2%
0.55	132	411	35	8	92.7%	92.2%	94.3%	79.0%
0.60	131	411	35	9	92.5%	92.2%	93.6%	78.9%
0.65	131	412	34	9	92.7%	92.4%	93.6%	79.4%
0.70	130	412	34	10	92.5%	92.4%	92.9%	79.3%
0.75	130	412	34	10	92.5%	92.4%	92.9%	79.3%
0.80	128	413	33	12	92.3%	92.6%	91.4%	79.5%
0.85	127	413	33	13	92.1%	92.6%	90.7%	79.4%
0.90	124	415	31	16	92.0%	93.1%	88.6%	80.0%
0.95	122	417	29	18	92.0%	93.5%	87.1%	80.8%

**Table 5:** Classification performance on the validation set using different evaluation indexes.

	AUC	ACC	Recall	Spe	F1 score	Youden index
Validation set	98.6%	93.0%	92.0%	94.1%	93.2%	0.813

## Data Availability

The data used to support the findings of this study are available from the corresponding author upon request.
